# Screening and Genomic Analysis of Alkaloid-Producing Endophytic Fungus *Fusarium solani* Strain MC503 from *Macleaya cordata*

**DOI:** 10.3390/microorganisms12061088

**Published:** 2024-05-27

**Authors:** Xinhong Wu, Nazidi Ibrahim, Yili Liang, Xueduan Liu

**Affiliations:** Key Laboratory of Biometallurgy of Ministry of Education, School of Minerals Processing and Bioengineering, Central South University, Changsha 410083, China; 235601001@csu.edu.cn (X.W.); nazidiibrahim1@csu.edu.cn (N.I.)

**Keywords:** *Macleaya cordata*, sanguinarine, chelerythrine, *Fusarium solani*, genomic analysis

## Abstract

The extensive harvesting of *Macleaya cordata*, as a biomedicinal plant and a wild source of quaternary benzo[c]phenanthridine alkaloids, has led to a rapid decline in its population. An alternative approach to the production of these bioactive compounds, which are known for their diverse pharmacological effects, is needed. Production of these compounds using alkaloid-producing endophytic fungi is a promising potential approach. In this research, we isolated an alkaloid-producing endophytic fungus, strain MC503, from the roots of *Macleaya cordata*. Genomic analysis was conducted to elucidate its metabolic pathways and identify the potential genes responsible for alkaloid biosynthesis. High-performance liquid chromatography (HPLC) and liquid chromatography–mass spectrometry (LC–MS) analyses revealed the presence and quantified the content of sanguinarine (536.87 μg/L) and chelerythrine (393.31 μg/L) in the fungal fermentation extract. Based on our analysis of the morphological and micromorphological characteristics and the ITS region of the nuclear ribosomal DNA of the alkaloid-producing endophyte, it was identified as *Fusarium solani* strain MC503. To the best of our knowledge, there is no existing report on *Fusarium solani* from *Macleaya cordata* or other medicinal plants that produce sanguinarine and chelerythrine simultaneously. These findings provide valuable insights into the capability of *Fusarium solani* to carry out isoquinoline alkaloid biosynthesis and lay the foundation for further exploration of its potential applications in pharmaceuticals.

## 1. Introduction

The main sources of natural drugs (biomedicines) are plants, often referred to as medicinal plants [1]. Alkaloids are one of the broad groups of naturally existing organic substances, comprising most of the significant groups of phytochemicals [2]. Isoquinoline alkaloids, especially sanguinarine and chelerythrine, exhibit a diverse range of biological activities and have attracted significant attention due to their potential therapeutic applications. They are among the major bioactive components of *Macleaya cordata* (*M. cordata*) [3,4]. Academics and industrial researchers developed a strong interest in these substances because of their significant antimicrobial and animal growth-promoting activities [5,6]; parasiticidal effects [7]; anti-inflammatory effects [8]; and anti-tumor activities [9]. Recently, there has been a steady rise in the daily demand for this particular plant due to its significant commercial worth. To date, wild and cultivated plants remain the major source of alkaloids for commercial use, but extracting these alkaloids involves time-consuming and labor-intensive processes. Increasing labor costs will surely raise the price of alkaloid production. Moreover, the extensive harvesting of wild resources of *M. cordata* has resulted in a decrease in its population [10]. In light of this, the development of a new viable, stable, sustainable, and industrial production system through microbial biosynthesis could be a promising alternative.

Endophytism is a process in which microbes form a mutualistic relationship with plants by residing in the plant’s tissues without exhibiting any disease-related symptoms. Therefore, endophytes represent a complex community of microorganisms that asymptomatically live in healthy, living plant tissues. The mutualistic interactions of endophytes and their host plants have a significant impact on the well-being of their hosts, both directly and indirectly [11]. They improve their responses to various biotic [12] and abiotic stresses [13]. In addition to being a priceless biological resource, through the synthesis of phytohormones [14], enhancement in photosynthetic activity [15], nitrogen fixation [16], mineral solubilization [16], and siderophore production [17], they also protect plants from disease by colonizing disease-causing regions, competing with pathogens for the present nutrients, producing antimicrobials, enhancing the plant’s immune system to counter phytopathogens, and triggering resistance mechanisms [18]. Furthermore, it is now well known that endophytes can produce medicinally significant “phytochemicals”, formerly thought to be solely synthesized by their host plants [19,20,21]. The ability of endophytes to biosynthesize similar or the same important secondary metabolites as their host plant plays a significant role in reducing the utilization of rare and/or endangered biomedicinal plants [22]. Furthermore, these substances have been found to exhibit antimicrobial, antiparasitic, anticancer, anthelmintic, antidiabetic, insecticidal, and anti-inflammatory activities [21,23].

The blueprint of an organism is encoded in its genome. Thus, genome mining has become a potent technique for exploring natural products [24,25,26] as well as improving their content through genetic modification [27] and is particularly suitable for endophytes that are rich sources of highly valued secondary metabolites derived from their host plant but are not amenable to versatile basic research techniques [28]. The genomics study of camptothecin-producing endophytes *Fusarium solani* and *Alternaria burnsii* NCIM 1409 associated with *Camptotheca acuminata* shed light on the genes and biosynthetic pathways involved in those processes [29,30]. Therefore, exploring the whole genome of endophytes provides valuable insights into the evolution and genetic basis of alkaloid production, whereby the specific genes and pathways involved in the biosynthesis of alkaloids can be identified and well understood. Additionally, with the rapid development of this emerging technology, novel endophytes with great potential and many effective pharmacological substances can be identified. Tentative studies of alkaloid-producing endophyte genomes to screen functional genes related to alkaloid biosynthesis, enzymes, steps, and intermediates can be used to understand the mechanisms underlying alkaloid production, optimize their production process, and genetically or metabolically alter the biosynthesis pathways in order to produce novel alkaloids with desired properties.

Herein, we report an alkaloid-producing endophytic fungus, *Fusarium solani* strain MC503, from the root of *M. cordata.* HPLC and LC–MS techniques were used for the qualitative and quantification of the alkaloid content in endophyte fermentation broth extract. In addition, whole-genome sequence analysis was carried out, and the genome of the strain was annotated in four public databases (Non-Redundant Protein Sequence (Nr), Gene Ontology (GO), Clusters of Orthologous Genes (COG), and the Kyoto Encyclopedia of Genes and Genomes (KEGG)) to understand the underlying metabolic pathways and genes responsible for alkaloid production by this strain.

## 2. Materials and Methods

### 2.1. Morphological Identification of the Isolate

After surface disinfection using 70% absolute ethanol and 2.5% sodium hypochlorite of *M. cordata* root grown in Liuyang City, Hunan Province (28°20′ N, 113°30′ E), endophytic fungi were isolated using cross-sectional culture and tissue-grinding gradient dilution plating methods. The growth conditions of endophytic fungi on the inoculated plates were observed for 15 consecutive days. Based on the different morphological appearances of endophytic fungal colonies, newly grown single colonies were observed to transfer into fresh culture medium every day [31]. Twenty strains of endophytic fungi were isolated from the root of *M. cordata.* The selection of strain MC503 was based on its ability to produce target alkaloids after HPLC and LC–MS validation experiments. It was cultivated on potato dextrose agar (PDA) plates at 30 °C (±1) for 7 days. The morphological characteristics such as color, shape, viscosity, and other colony growth features were observed, and the micromorphological features such as the shape of spores, structures of conidiophores, and appearance of hyphae were observed using an electron microscope (DV1200, CNOPTEC. Co., Ltd., Chongqing, China).

### 2.2. Fungal DNA Extraction, PCR Amplification of ITS, and Sanger Sequencing

The endophytic fungus was cultured in a 300 mL flask containing 100 mL of potato dextrose broth (PDB) at 30 °C (±1) for 7–10 days [31]. For DNA extraction, about 1.5 mL of fungal liquid culture solution was used to extract the total genomic DNA using fungi gene DNA extraction test kits (centrifugal column type (B027002023)) from Beijing Biotech Biotechnology Co., Ltd., strictly according to the manufacturer’s instructions. A NanoDrop ND-83 1000 spectrophotometer (NanoDrop Technologies, Wilmington, NC, USA) was used to determine the purity and concentration of DNA. The extracted DNA sample was subjected to a polymerase chain reaction (PCR) of the internal transcribed spacer (ITS) region of rDNA using universal primers ITS1 (5′-TCCGTAGGTGAACCTGCGG-3′) and ITS4 (5′-TCCTCCGCTTATTGATATGC-3′) [32]. The PCR reaction was performed in a 20 μL final volume containing 0.5 μL of template DNA, 1.0 μL of forward and reverse primer, 2.0 μL of 10× Ex Taq buffer, 0.2 μL of 5u Ex Taq, 1.6 μL of 2.5 mM dNTP Mix, and 13.7 μL of deionized water. The amplification conditions were as follows: denaturation at 95 °C for 5 min followed by 30 cycles of annealing at 95 °C for 30 s, 56 °C for 30 s, 72 °C for 1 min 30 s, and elongation at 72 °C for 10 min [33]. The quality of the amplified product was verified on an agarose gel by running at 5 V/cm for 20 min. The PCR amplicon was purified using a SanPrep Column DNA Gel Extraction Kit (Sangon Biotech, Shanghai, China), and the product was sequenced by the Sanger sequencing approach at Sangon Biotech Co. Ltd. (Changsha, China). The quality of the sequences received was checked by viewing the chromatogram on Chromas 2 (V. 2.6.6), and the identity of the endophyte was confirmed by blasting the checked sequence against the NCBI database using BLASTn [34]. The ITS sequences of the MC503 strain, high-similarity strains, and type strain were aligned by ClustalW. The phylogenetic tree was constructed with MEGA 11 software [35] using the neighbor-joining (NJ) method and Kimura two-parameter model. Bootstrap support showed that the value was based on 1000 replications. Subsequent genome sequencing and bioinformatics analysis were conducted using the filtered strain.

### 2.3. HPLC Sample Preparation and Detection Conditions

The fungal isolates were cultured in potato dextrose broth at 30 °C (±1) in a 180 rpm shaking incubator for 10 days. Then, 40 mL of the fermentation broth was collected using medical gauze and poured into 50 mL conical flasks. The pH was adjusted to alkaline (9–11) using concentrated sodium hydroxide. The extraction was carried out at a 2:1 ratio (fermentation broth volume–ethyl acetate). The mixture was shaken and left for 12 h to achieve the desired extraction effect. The organic phase was collected and evaporated using a rotary evaporator. The extract was then obtained by dissolving the residues in 2–3 mL of methanol and filtering 1–1.5 mL of the extract through a 0.22-microfilter membrane into the chromatographic bottle for detection.

The four alkaloid standard products (sanguinarine, chelerythrine, allocryptopine, and protopine) were prepared into a mixed standard solution of 0.2 mg/mL. The mixed standard solution of the four alkaloids was diluted into a series of six standard reference solutions with concentrations of 200 µg/mL, 100 µg/mL, 50 µg/mL, 25 µg/mL, 12.5 µg/mL, and 6.25 µg/mL. The detection conditions for HPLC were set as follows: mobile phase: A (acetonitrile) and B (0.1% formic acid water); flow rate: 1.0 mL/min; injection volume: 10 µL; column temperature: 35 °C; gradient elution procedure: 0–11 min, 75% B; 11–27 min, 75–40% B; 27–29 min, 40–75% B; 29–40 min, 75% B; detection wavelength: 284 nm; YMC-Triart C18 column (250 nm × 4.6 nm, 5 µm).

### 2.4. LC–MS Sample Preparation and Detection Conditions

The same fermentation and extraction method for HPLC was adopted for LC–MS samples. The detection conditions for LC–MS were set as follows: Analysis was carried out using Agilent ultra-high-performance liquid chromatography coupled with quadrupole time-of-flight mass spectrometry (UPLC–Q–TOF–MS). LC separations were accomplished using an Agilent HXQ–C18–214 column (Santa Clara, CA, USA) (2.1 mm × 150 mm) at 40 °C. A flow rate of 0.3 mL/min was chosen, and 0.1% formic acid water (A) and acetonitrile (B) made up the mobile phase. The gradient elution program was as follows: 0~9.0 min, 0–10% B; 9.0~11.50 min, 10–21% B; 11.50~15 min, 21–43% B; 15~28 min, 43–85% B; 28~30 min, 85–90% B; 30~33 min, 95–95% B; 33~33 min, 95–10% B; and 33~35 min, 10–10% B. The injection volume was 4.0 µL.

### 2.5. Genome Sequencing, Assembly, and Functional Annotation

The sequencing work was conducted at Shanghai Meiji Biomedical Technology Co., Ltd. (Majorbio BioTech Co., Shanghai, China). The sequencing was performed using the Illumina HiSeq 2000 sequencing platform, generating no less than 100× of 150 bp paired-end (PE150) sequencing data. For a sufficient sequencing depth required for the assembly of the genome, approximately 1.4 GB of data were generated from the sequencing. Contaminants, namely adapter sequences; bases containing non-A, G, C, and T at the 5’ end; 10% N-containing reads; and low-quality reads, were removed to clean the reads generated using Fastp (version 0.20.0) [36]. The sequence was then assembled using the default parameters of SOAPdenovo2 (v2.04) [37], and GapCloser (v1.12) was used to fill gaps in the assembly results [37]. The completeness of the assembled genome was evaluated using BUSCO (v5.4.5) and CEGMA (v2.0) [38,39]. MAKER2 (v2.32) was used for gene prediction [40]. Barrnap (v0.9) and tRNA-scan-SE (v2.0.12) software were used to predict the rRNA and tRNA contained in the genome. Functional annotation was executed using Diamond (v0.8.35) [41] and BLAST+ (v2.3.0) in different public databases, including the NR database (http://ftp.ncbi.nlm.nih.gov/blast/db/, accessed on 20 November 2023), Eggnog database for COG classification (http://eggnogdb.embl.de/#/app/home, accessed on 20 November 2023) [42], GO database (https://geneontology.org/, accessed on 20 November 2023) [43], and KEGG database (http://www.genome.jp/kegg/, accessed on 20 November 2023) [44]. 

## 3. Results

### 3.1. Morphological Characteristics of the Isolate

**Figure 1:**
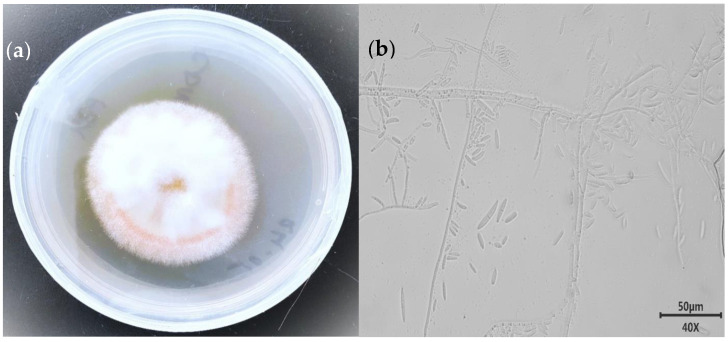
Morphological appearance of the strain: (**a**) front view of five-day-old culture and (**b**) unstained septate macro-conidiation mycelium under an ×40 microscopic objective lens.

To observe the morphological and micromorphological characteristics of the strain, the isolate MC503 was cultured on a PDA plate at 30 °C (±1) for 7 days. The colonies of strain MC503 were initially white to light red in age and cottony form and grew fast to attain a diameter of more than 5 cm in five days, also featuring a pale yellow-red background color (Figure 1a). Under the environmental-scanning electron micrograph, the mycelium showed typical unstained septate simple and branched macroconidia as well as single-celled and clavate microconidia (Figure 1b). The isolated fungus was initially identified as a member of the *Fusarium solani* species based on the cultural traits and microscopic appearance of the growth according to the previous report on the *Fusarium solani* identification [45]; however, further molecular approaches were required for accurate identification of the fungus at the genus and species levels.

**Figure 2:**
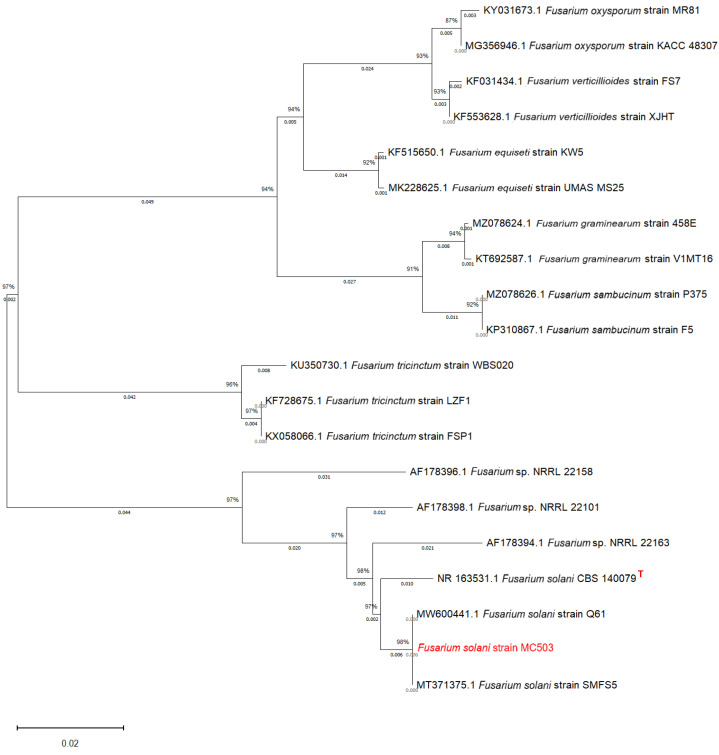
Phylogenetic tree of the strain showing its evolutionary relationship based on rDNA-ITS sequences. The numbers at the nodes indicate the levels of bootstrap support based on the kimura 2-parameter model and neighbor-joining method. Bootstrap support showed that the value was based on 1000 replications. The top right corner of the type strain was marked with a red “T”.

The nucleotide sequence of the ITS region of MC503 sample was amplified and sequenced, obtaining 565 bp. After the sequence edition, a nucleotide blast was performed in the NCBI Genebank database, and it was found that MC503 had high sequence similarity with the *Fusarium solani* strain Q61 (GenBank accession number MT371375.1) and *Fusarium solani* strain SMFS5 (GenBank accession number MW600441.1). The percentage identity, coverage, and sequence length for *Fusarium solani* strain Q61 were 100%, 100%, and 569 bp, respectively, and those of *Fusarium solani* strain SMFS5 were 100%, 100%, and 571 bp, respectively. *Fusarium solani* CBS140079 (GenBank accession number NR163531.1) was found to be the type strain. The sequence of the strains with high similarity and type strain was downloaded and aligned with the MC503 strain sequence using MEGA 11 software. The phylogenetic tree was constructed using the neighbor-joining 1000 bootstraps. The neighbor-joining algorithm inferred the evolutionary history of the species (Figure 2). Among them, MC503 had the highest sequence similarity to *Fusarium solani* species; it exhibited 98% sequence identity to Fusarium solani strain Q61 and Fusarium solani strain SMFS5. Based on the morphological, micromorphological, and ITS sequence analysis results, the cultured endophytic strain was confirmed to be *Fusarium solani* and was named *Fusarium solani* strain MC503. 

### 3.2. HPLC/LC–MS

**Figure 3:**
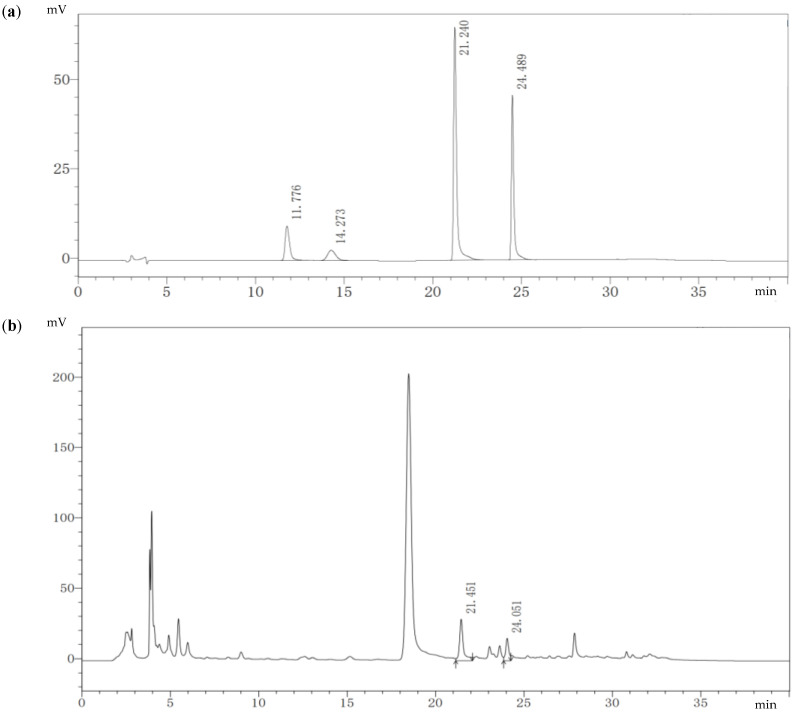
HPLC results: (**a**) four-reference standard solution of alkaloid showing tR of protopine, allocryptopine, sanguinarine, and chelerythrine, and (**b**) endophyte fermentation broth extract showing tR of sanguinarine and chelerythrine.

The fermentation extract of strain MC503 was the only sample observed to contain the target alkaloids after verification experiments. The HPLC results show that reference protopine, allocryptopine, sanguinarine, and chelerythrine had strong absorption peaks near the retention time (tR) of 11.776 min, 14.273 min, 21.240 min, and 24.489 min, respectively (Figure 3a). The regression equation of the standard curves between peak area and concentration of reference standards is shown in Figure S1, where the correlation coefficient (R^2^) of protopine has a value of 0.9982 (Figure S1a); allocryptopine, 0.9995 (Figure S1b); sanguinarine, 0.9996, (Figure S1c); and chelerythrine, 0.9998, (Figure S1d), with linearity within 6.25–200 µg/mL. The fermentation extracts of the strain MC503 when cultured in 100 mL of PDB medium at 30 °C (±1) in a 180 rpm shaking incubator for 10 days had absorption peaks at tR of 21.451 min and 24.051 min (Figure 3b), which are similar to sanguinarine and chelerythrine reference standard peaks tR, indicating the production of these two alkaloids by this strain. The concentrations of sanguinarine and chelerythrine in the fermentation broth of the strain were about 536.87 µg/L and 393.31 µg/L, respectively, calculated using the regression equation of the standard curves.

**Figure 4:**
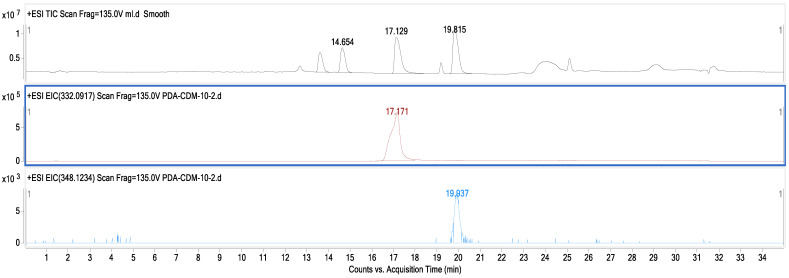
LC–MS result displaying chromatograms of the reference standard (in black color) and *Fusarium solani* strain MC503 fermentation extract showing the presence of sanguinarine (in brown color) and chelerythrine (in blue color).

**Figure 5:**
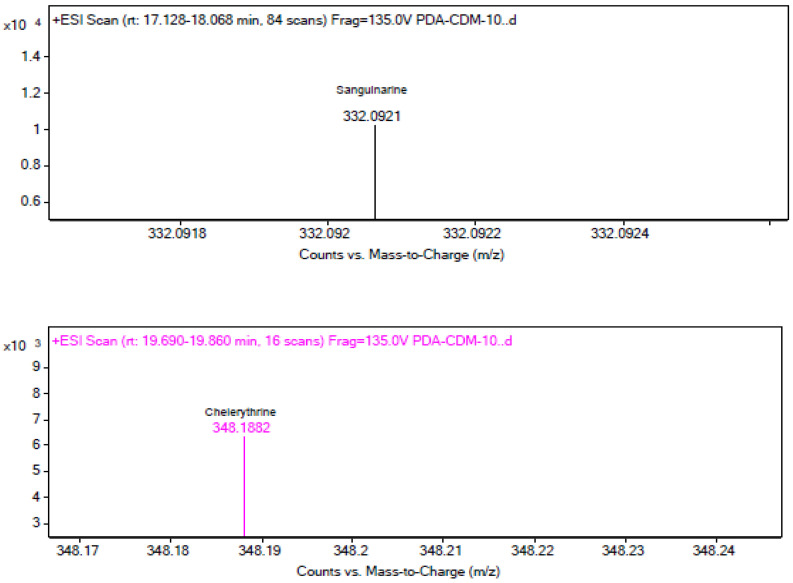
LC–MS results displaying *m*/*z* values of sanguinarine (in black color) and chelerythrine (in purple color) from *Fusarium solani* strain MC503 fermentation extract.

The LC–MS results shown in Figure 4 display three layers of graphs distinguished by different colors. The reference standards sanguinarine and chelerythrine chromatograms with tR values of 17.129 and 19.815 are presented in black, while the chromatograms in brown and blue colors on the second and third layers of the graph are the results of the fermentation broth extract of strain MC503. Notably, the peaks at 17.171 min (in brown) and 19.937 min (in blue) align with the reference standards sanguinarine and chelerythrine (in black color), providing strong evidence for the production of these two metabolites by the strain. In addition, Figure 5 presents the *m*/*z* values of the two metabolites (332.0921 and 448.1882) for sanguinarine (in black) and chelerythrine (in purple), respectively, from the LC–MS results of strain MC503 fermentation broth extract analyzed using Agilent Masshunter qualitative analysis software. Moreover, no alkaloid was detected in the blank control of both HPLC and LC–MS analyses. According to the results of HPLC and LC–MS analyses, we inferred that the endophytic fungus *Fusarium solani* strain MC503 has the potential to biosynthesize sanguinarine and chelerythrine, similar to its host plant [3,46].

### 3.3. High-Quality Genome Sequencing, Assembly, and Functional Annotation of Fusarium solani Strain MC503

Following the quality control assessment using Fastp (v0.20.0) and assembly of the *Fusarium solani* strain MC503 genome sequencing data, we successfully obtained the complete genome content of *Fusarium solani* strain MC503. The quality control assessments, assembly, and annotation results are displayed in Table 1. A total of 1,875,102 high-quality single clean reads and 16,367,452 × 2 clean paired reads with a total of 5,199,691,508 clean bases (bp) were obtained. The clean reads exhibited a high level of quality, with a clean Q20 of 97.48% and a clean Q30 of 92.45% after quality control. These scores demonstrated the consistency and accuracy of the obtained sequencing data; the details can be seen in the Supplementary Data Sheet S1. By using BUSCO and CEGMA software for genome assembly quality assessment, a genome size of 61,238,035 bp of strain MC503 was generated from 3747 contigs, with a total base of 61,176,316 bp and a largest contigs’ length of 878,995. The quality of the contigs was also evaluated using Contig N_50_ and Contig N_90_, found to be 199,899 bp and 10,370 bp, respectively, with a G+C content of 50.651% (Figure S2; Supplementary Data Sheet S2). The results of the genome quality assessment indicated the completeness and accuracy of the genome assembly of the strain MC503, as the BUSCO result merged 98.8% and CEGMA 97.18%.

A total of 17,668 coding sequences (CDSs) with an average length of 1871.31 bp and a total length of 33,062,279 bp were identified using MAKER2 (v2.32) software. The non-coding RNA comprised 69 ribosomal RNA (rRNA) sequences and 359 transfer RNA (tRNA) sequences identified from the strain genomic data. Moreover, the 17,668 protein-coding sequences were annotated in multiple functional public databases based on sequence similarity comparison. About 17,074 (96.64%) of the protein-coding genes sequences in strain MC503 matched the genomic data in the Non-Redundant Protein Sequence (Nr) database. The Nr database has the highest annotation rate among all the employed databases, which shows the global abundance of the strain and reflects its significant advantage in commercial use. Furthermore, 10,895 (61.67%) protein-coding genes were annotated in the Gene Ontology (GO) database, which was functionally assigned to three main classes: “cellular component” (6347), “molecular function” (8491), and “biological process” (4843).

**Figure 6:**
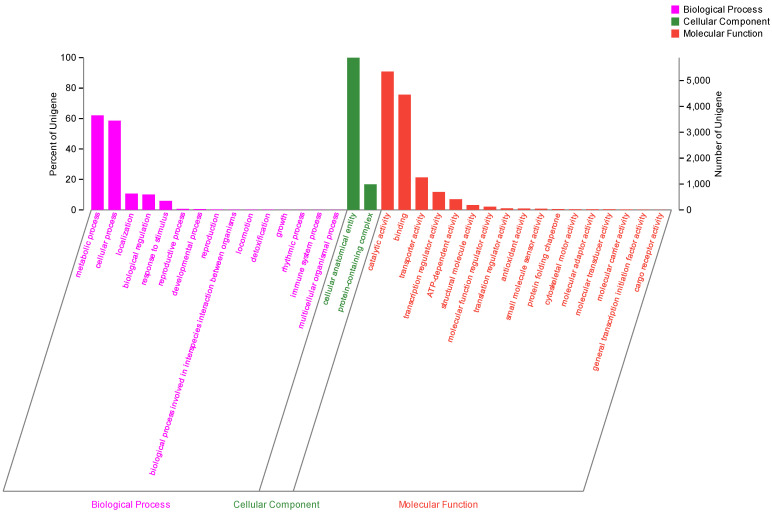
Gene Ontology functional annotation of the *Fusarium solani* strain MC503.

The “cellular anatomical entity” (5887), “catalytic activity” (5351), and “binding” (4456) were the top three most enriched terms. However, the metabolic process was the fourth one with 3656 annotated coding genes, which are directly related to the biosynthesis of secondary metabolites, including alkaloids (Figure 6).

**Figure 7:**
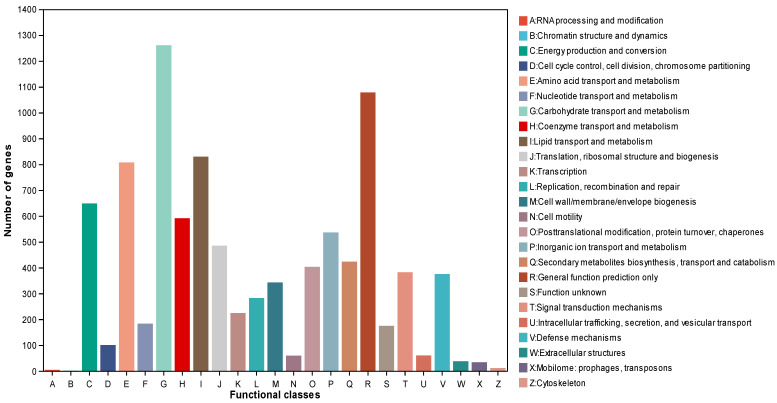
Cluster of Orthologous Groups functional annotation of the *Fusarium solani* strain MC503.

Further functional annotation analysis using the Clusters of Orthologous Genes (COG) database annotated 7988 (45.21%) genes, which showed that “carbohydrate transport and metabolism” (G, 1261) had the highest number of genes, followed by “general function prediction only” (R, 1078), “lipid transport and metabolism” (I, 829), and “amino acid transport and metabolism” (E, 807) (Figure 7). The COG annotation results displayed the great potential of strain MC503 in the biosynthesis of diverse secondary metabolites, as all the functional groups with higher annotated genes are directly or indirectly related to secondary metabolite biosynthesis, including alkaloids. Moreover, annotation of 5769 coding genes using the Kyoto Encyclopedia of Genes and Genomes (KEGG) database showed the genes were enriched in pathways belonging to six categories (Figure S3): metabolism (7955), human disease (2621), organismal system (1222), genetic information processing (964), environmental information processing (820), and cellular processes (1028), in which most genes were enriched in pathways of global and overview maps, amino acid metabolism, and carbohydrate metabolism. The KEGG annotation result indicated that carbohydrate metabolism and amino acid metabolism were the second and third most enriched categories among the observed KEGG metabolic pathways, with 650 and 580 annotated genes, respectively. This further highlights the strain’s substantial involvement in metabolic activities and the synthesis of secondary metabolites.

### 3.4. Sequence Similarity of KEGG-Genes Encoding Enzymes Involved in Sanguinarine and Chelerythrine Biosynthesis against Strain MC503 Genome

Isoquinoline alkaloids are tyrosine-derived plant alkaloids with an isoquinoline skeleton. Among them, quaternary benzophenanthridine alkaloids form an important group with potent pharmacological activities, which include sanguinarine and chelerythrine. Table S1 presents the similarities between the enzyme reference sequences involved in the alkaloid biosynthesis pathway from tyrosine to sanguinarine and chelerythrine from the KEGG database and the *Fusarium solani* strain MC503 genome sequences using the genomic target fragment finder tool. Among the enzymes reported to catalyze sanguinarine and chelerythrine biosynthesis in the strain host plant, some were found to have sequence similarities in the strain MC503 genome, which include tyrosine decarboxylase (TYDC (76.5%)), (RS)-norcoclaurine 6-*O*-methyltransferase (6OMT (31.9%)), (RS)-1-benzyl-1,2,3,4-tetrahydroisoquinoline *N*-methyltransferase (CNMT (63.3%)), *N*-methylcoclaurine 3′-monooxygenase (CYP80B1, NMCH (64.7%)), cheilanthifoline synthase (CYP719A14, CFS (64.7%)), (*S*)-stylopine/(*S*)-canadine synthase (SCS, STS (57.9%)), methyltetrahydroprotoberberine 14-monooxygenase (CYP82N4, TNMT (25.5%)), protopine 6-monooxygenase (CYP82N2_3, P6H (35.3%)), and tetrahydroprotoberberine oxidase/dihydrobenzophenanthridine oxidase (STOX, DBOX (31.9%)). However, among the reported enzymes, (*S*)-norcoclaurine synthase (NCS), 3′-hydroxy-*N*-methyl-(*S*)-coclaurine 4′-*O*-methyltransferase (4OMT), reticuline oxidase/berberine bridge enzyme (BBE), and methyltetrahydroprotoberberine 14-monooxygenase (MSH) were not found to have any sequences similarities with the strain genome. These may hypothetically indicate the utilization of distinct biosynthetic pathways by the strain.

### 3.5. Possible Metabolic Pathways of Alkaloid Production by the Strain MC503

**Figure 8:**
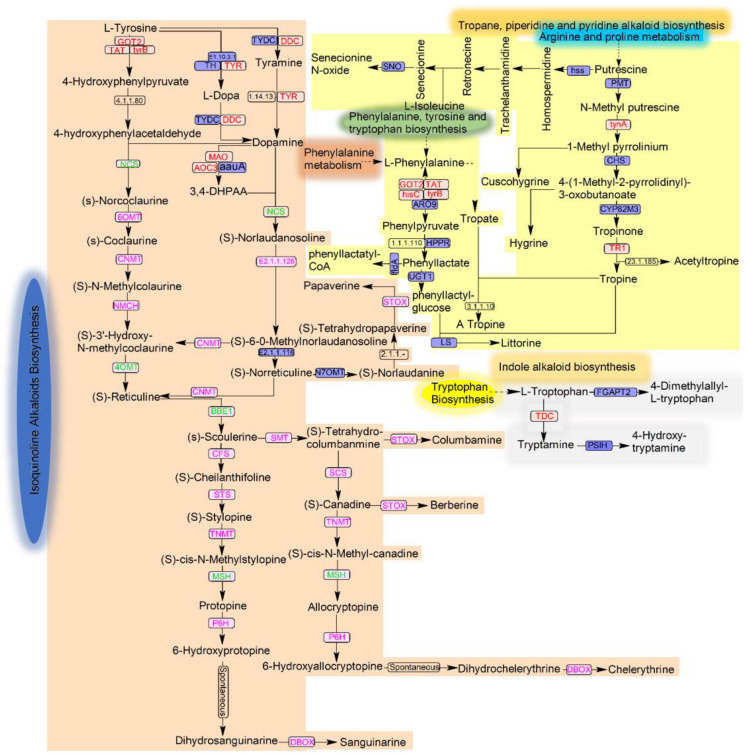
Alkaloid biosynthesis pathways: isoquinoline alkaloid is shown in a light brown background color, indole alkaloid in a light ash color, and tropane, piperidine, and pyridine in light yellow. The enzymes found in the genome sequences of the strain based on KEGG annotation are presented in red; the sanguinarine and chelerythrine biosynthesis enzymes queried against the strain’s genome are in pink; and the not-found and unstudied enzymes are in green and black, respectively.

Isoquinoline, indole, and tropane alkaloids are specific types of alkaloids that are synthesized through distinct biosynthetic pathways. The amino acids phenylalanine, tyrosine, and tryptophan are the precursor amino acids for the synthesis of these alkaloids. The genes encoding some of the important enzymes in these alkaloid biosynthetic pathways were annotated in the strain MC503 genome using the online KEGG database; as such, the possible metabolic pathways of these alkaloid productions were predicted (Figure 8 and Figure S4). The genes encoding upstream enzymes of isoquinoline alkaloid biosynthesis: aspartate aminotransferase (GOT2), tyrosine aminotransferase (TAT), aromatic-amino-acid transaminase (tyrB), tyrosinase (TRY), aromatic-L-amino-acid decarboxylase (DDC), monoamine oxidase (MAO), and primary-amine oxidase (AOC3, AOC2, tynA), responsible for converting tyrosine to 4-hydroxyphenylpyruvate, Tyramine, L-Dopa, dopamine, 4-hydroxyphenylacetaldehyde, and 3,4-DHPAA, respectively, were initially annotated in the KEGG database from the genome of strain MC503 and are indicated in red color in Figure 8. Subsequently, the genes encoding the enzymes 6OMT, CNMT, and NMCH catalyzing the intermediates (*S*)-Norcoclaurine, (*S*)-coclaurine, (*S*)-*N*-Methyl colaurine (*S*)-Coclaurine, (*S*)-3′-Hydroxy-*N*-methylcoclaurine, (*S*)-6-*O*-Methyl Norlaodanosoline, and (*S*)-*N*orreticuline, (*S*)-reticuline to scoulerine from 4-hydroxyphenylacetaldehyde, and 3,4-DHPAA were found in the strain genome via sequence similarity analysis. However, the genes encoding the enzymes NCS, 4OMT, and BBE were absent in the strain MC503 genome.

Nonetheless, the sequence similarity analysis also observed the genes encoding the enzymes involved in the downstream biosynthesis of sanguinarine and chelerythrine alkaloids. The obtained gene enzyme sequences were CFS, STS/SCS, TNMT, P6H, and STOX/DBOX, which catalyzed the intermediate (*S*)-scoulerine to the two mentioned byproducts, as shown in Figure 8. However, another enzyme MSH was not found in the downstream process. Notably, as the sanguinarine and chelerythrine were obtained, this indicated the potential of the strain to biosynthesize these end products in the absence of these enzymes, which indicated its distinct metabolic pathways of isoquinoline alkaloid biosynthesis from its host plant (though they are likely the same).

In expanding the potential capabilities of strain MC503 in synthesizing other alkaloid compounds based on genome analysis in the KEGG database, we found the first important key enzyme, tryptophan decarboxylase (TDC), in the strain genome, which is responsible for catalyzing L-tryptophan to tryptamine—the first bioconversion in the biosynthesis of indole alkaloids. Moreover, in tropane alkaloid biosynthesis, the enzymes tynA and TRT that catalyze the bioconversion of *N*-methyl putrescine to 1-methyl pyrrolinium, and tropinone to tropine, respectively, were observed. Additionally, the four enzymes (GOT2, TAT, hisC, and tyrB) initiating the biosynthesis of tropane alkaloids from phenylalanine, tyrosine, and tryptophan biosynthesis were found (Figure 8). These indicated the possibility of the strain’s involvement in indole and tropane alkaloid biosynthesis. Detailed information, including KO ID, KEGG ID, and KO description; pathway map ID; and the whole biosynthesized alkaloid compounds of these candidate genes, is available in Table S2 and Supplementary Data Sheet S3.

## 4. Discussion

Since the discovery of the taxol-producing endophytic fungus *Taxomyces andreanae* [47], the bioactive compounds derived from fungal endophytes have attracted pharmacological research attention. Our lab has successfully isolated fungal endophytes from a variety of naturally occurring medicinal plants, such as *Ginkgo biloba* L. [48], with the capability of producing plant natural products comparable to their host plants. Employing endophytic fungi fermentation to produce natural bioactive compounds is an effective and reliable strategy when proper technological advancement is used in mediating the capability of these entities. There are numerous reports on endophytic *Fusarium*, including its pathogenicity to plants and animals [49,50,51], as well as its ability to synthesize host bioactive compounds through long-term symbiosis with medicinal plants [20,31,52]. Nonetheless, to the best of our knowledge, this is the first report that *Fusarium solani* obtained from *M. cordata* roots has the ability to produce both sanguinarine and chelerythrine simultaneously. Endophytic fermentation represents an innovative and sustainable approach to the production of alkaloids, significantly diminishing the reliance on direct extraction methods from wild *M. cordata*. This bioprocess offers a myriad of advantages over the traditional extraction of bioactive compounds from plants, including reduced timeframes, simplified cultivation requirements, and an eco-friendly stance that aligns with green initiatives. Compared to the direct construction of chassis cells to produce alkaloids, endophytic fungi that produce alkaloids obtained from *M. cordata* tissue may have undergone gene transfer with their host under conditions of long-term symbiosis. These fungi inherently possess a suite of genes that are responsible for the synthesis of plant secondary metabolites. Upon activation of these gene clusters, they can also open up a new avenue for the discovery of novel compounds.

In this research, we isolated endophytic fungi from *M. cordata* roots and tested their ability to produce alkaloids as their host plant. Fortunately, we found that sample *Fusarium solani* strain MC503 has the capability of biosynthesizing sanguinarine and chelerythrine. The results of HPLC and LC–MS analysis in this report have shown the potentiality of this strain in producing these significant bioactive compounds as its host plant. The yields of sanguinarine and chelerytherine were 536.87 µg/L and 393.31 µg/L, respectively. A significant number of researchers have reported similar findings. Sanguinarine-producing endophytic *Fusarium proliferatum* strain BLH51, an endophytic fungus isolated from the leaves of *M. cordata* grown in Dabie Mountain, China, was reported to produce a quantified amount of 178 µg/L of sanguinarine upon fermenting in potato dextrose liquid medium for 10 days at 28 °C and 200 rpm [31]. The Smolke CD research team found that the titers of protopine and sanguinarine in engineered yeast were 252 μg/L and 80 μg/L, respectively [53]. In our investigation, we discovered that the alkaloid yield from *Fusarium solani* strain MC503 surpasses the levels documented in prior research. This finding suggests that *Fusarium solani* strain MC503 possesses considerable potential for the development and production of alkaloids, which can be instrumental in various sectors including pharmaceuticals, chemical industry, and veterinary science.

Proverbially, the complete biosynthetic pathways of numerous significant benzylisoquinoline alkaloids, including chelerythrine and sanguinarine, have been elucidated, and their heterologous productions in microbial hosts have been recognized [54]. However, their production titers frequently fall in the range of micrograms to a milligrams per liter and cannot reach levels sufficient for commercial production (>1 g/L) [55]. The in vitro biosynthesis of secondary metabolites by endophytes is regulated by several factors, most of which are not fully understood. Some common factors include the composition of culture media, nutritional ratio, aeration, pH, temperature, light, precursor substances, inducing factors, and the period of fermentation or cultivation [56], which lead to variation and unstable production of such important products during subsequent culturing of endophytes independent of their host [29]. Utilizing shaking flasks for the cultivation of endophytic fungi, the nutritional content provided by the culture medium is inherently constrained, potentially representing a factor in the restricted biosynthesis of alkaloids. As previously reported, the shared biosynthetic pathway for sanguinarine and chelerythrine diverges at the stage of scoulerine [57]. Similarly, within the *Fusarium solani* strain MC503, two distinct synthetic pathways exist to separately produce sanguinarine and chelerythrine. This leads to a dispersion of the synthesis products, resulting in relatively low concentrations of sanguinarine and chelerytherine detected in the fermentation broth. Furthermore, although attempts to produce and isolate secondary metabolites on a small scale in the laboratory have occasionally been effective, efforts to use endophytes in large-scale industrial production have resulted in low yields due to a significant number of challenges. Therefore, to produce a significant quantity of secondary metabolites at an industrial scale, several strategies for enhancing the quantity and quality have been proposed, such as metabolic and genetic engineering, optimizing cultivation processes, co-culture fermentation, and other novel techniques. Some of the genetic modification approaches include random mutagenesis, genome shuffling, and gene overexpression to improve the expression of the desired products [58,59].

The genes encoding the enzymes tyrosine decarboxylase, tyrosinase, tyrosine aminotransferase, aromatic-amino-acid transaminase, monoamine oxidase, primary-amine oxidase, aspartate aminotransferase and other enzymes responsible for converting tyrosine to dopamine, tyramine, 4-hydroxyphenylacetaldehyde, and diverse group of alkaloids were annotated in the *Fusarium solani* strain MC503. The key intermediates, (*S*)-reticuline, are catalyzed by 6-*O*-methyltransferase (6OMT), cocoylline-*N*-methyltransferase (CNMT), *N*-methylcoclaurine hydroxylase (NMCH), 4-*O*-methyltransferase (4OMT), and berberine bridge enzyme (BBE) to convert into scoulerine [60,61]. Sanguinarine and chelerythrine are synthesized from scoulerine through different enzymes, including cheilanthifoline synthase (CFS)/(*S*)-scoreline 9-*O*-methyltransferase (SMT), stylopine synthase (STS)/(*S*)-canadine synthase (SCS), tetrahydroprotoberberine cis-*N*-methyltransferase (TNMT), (*S*)-cis-*N*-methylstylopine 14′-hydroxylase (MSH), protopine 6′-hydroxylase (P6H), and dihydrobenzphenanthridine oxidase (DBOX) [62,63,64]. The percentage identity, coverages, scores, and E-values presented in Table S1 indicate a level of similarity between the sequences of genes encoding the enzymes and the presence of these genes in the strain’s genome. However, although the genes encoding four of these enzymes (NCS, 4OMT, BBE, and MSH) are missing or not expressed by the *Fusarium solani* strain MC503, Figure S4 and Table S1, there is a possibility of bypassing or utilizing another metabolic route by the strain; the bioconversion may also be spontaneous. These findings need to be verified in further analyses. According to reports, (*S*)-cis-*N*-methylstylopine 14′-hydroxylase (MSH) can be bypassed by cocoylline-*N*-methyltransferase (CNMT), methyl transferase (MT), cyclooxygenase (COA), and oxidase to convert (*S*)-succinine into protopine and allocryptopine [62]. The genomic analysis findings strongly supported the detection of sanguinarine, and chelerythrine in the fermentation broth of *Fusarium solani* strain MC503 results based on HPLC, LC–MS. In our study, the results of HPLC and LC–MS showed that only sanguinarine and chelerythrine were detected, and no protopine and allocryptopine were detected. However, sanguinarine and chelerythrine are derived from the conversion of protopine and allocryptopine, respectively. As indicated by the results of functional gene annotation, the absence of the MSH may lead to the inability to synthesize protopine and allocryptopine. Alternatively, all protopine and allocryptopine can be converted into sanguinarine and chelerythrine [57,62,64]. Here, we are more concerned with whether there are other biosynthetic pathways in *Fusarium solani* strain MC503. As a widely studied and highly promising fungus, the genome research of *Fusarium* is relatively mature [65]. Many *Fusarium* have been used as sources of microbial strains for producing isoquinoline alkaloids [66]. Moreover, there is a significant need to further explore the potential of biosynthesizing a complete isoquinoline alkaloid consisting of completely diverse possible enzymes involved in biosynthetic pathways. The availability of this information could enlighten researchers in metabolically altering the pathway process or modifying enzyme expression to significantly achieve complete sanguinarine and chelerythrine production; it may also enhance production titers to meet industrial requirements. *Fusarium solani* strain MC503 which we screened from *M. cordata*’s root may also become a more high-yield functional strain through genetic modification.

## 5. Conclusions

Extracting relevant bioactive substances from medicinal plants is a costly and time-consuming process that involves the use of large quantities of several plant parts. Therefore, shifting from medicinal plants to endophytes for extracting similar plant-driven compounds independent of the host plant will greatly help in overcoming the challenges associated with direct extraction from the host. In this article, we screened an endophytic fungus associated with *M. cordata* root and studied its ability to biosynthesize some isoquinoline alkaloids as its host plant. The HPLC, LC–MS, and genomic analysis of the strain demonstrated its capacity to mimic its host in producing sanguinarine and chelerythrine simultaneously. Although the quantity of sanguinarine and chelerythrine reported in this research surpassed the previous reports, the production titer remained in micrograms. As such, priority needs to be given to genetic and molecular-based research for better comprehension of the mechanisms underlying their production and optimization processes using advanced technological approaches to improve the production titers.

## Figures and Tables

**Table 1:** *Fusarium solani* strain MC503 genomic assembly and annotation information.

Genomic Information	Predictive Data
Clean base	5,199,691,508
Q20 (%)	97.48
Q30 (%)	92.45
Genome size (bp)	61,238,035
Complete BUSCO (%)	98.8
Complete CEGMA (%)	97.18
Total No. of contigs bases (bp)	61,176,316
Contigs N_50_ (bp)	199,899
Contigs N_90_ (bp)	10,370
GC content (%)	50.651
Total CDS	17,668
CDS total length (bp)	33,062,279
CDS average length (bp)	1871.31
No. of tRNA	359
No. of rRNA	69
GO database	10,895 (61.67%)
Nr database	17,074 (96.64%)
COG database	7988 (45.21%)
KEGG database	5769 (32.65%)

## Data Availability

The data availability link and location will be provided later prior to online publication. The names of the repository/repositories and accession number can be found at: https://www.ncbi.nlm.nih.gov/bioproject/ PRJNA1114450, accessed on 20 November 2023.

## Supplementary Materials

The following supporting information can be downloaded at: https://www.mdpi.com/article/10.3390/microorganisms12061088/s1, Figure S1: HPLC standard curve of the four reference alkaloids showing the linearity of the four reference standards within the range of 200 µg/mL–6.25 µg/mL: (a) protopine, (b) allocryptopine, (c) sagunarine, and (d) chelerythrine; Figure S2: Guanine–cytosine content presence in the genomic data of the strain; Figure S3: KEGG database annotation showing the enrichment of coding genes in six categories of pathways, where metabolism has the highest number of annotated genes (7955), composed of amino acid metabolism (580), carbohydrate metabolism (650), etc.; Figure S4: Isoquinoline alkaloid biosynthetic pathways from the KEGG database showing annotated genes from strain MC503 genome; Table S1: Comparative analysis of sanguinarine and chelerythrine biosynthesis reference enzyme sequences against genome sequences of the Fusarium solani strain MC503; Table S2: Annotated enzymes and alkaloid profiles of Fusarium solani strain MC503; Supplementary Data Sheet S1: Strain MC503_Illumina_statistics data; Supplementary Data Sheet S2: Strain MC503 assembly summary; Supplementary Data Sheet S3: Candidate genes encoding enzymes responsible for key steps in the alkaloids’ biosynthetic pathways: subsheet1: Indole alkaloid genes; subsheet2: Isoquinoline alkaloid genes; subsheet3: Tropane alkaloid genes.
