# Supplementary material for: Screening and Genomic Analysis of Alkaloid-Producing Endophytic Fungus Fusarium solani Strain MC503 from Macleaya cordata
Source: Microorganisms. 2024 May 27;12(6):1088. doi: 10.3390/microorganisms12061088 (PMC11206080; doi:10.3390/microorganisms12061088)
Supplement: Supplementary file 1 [file microorganisms-12-01088-s001.zip › Supplementary Tables and Figures.pdf]

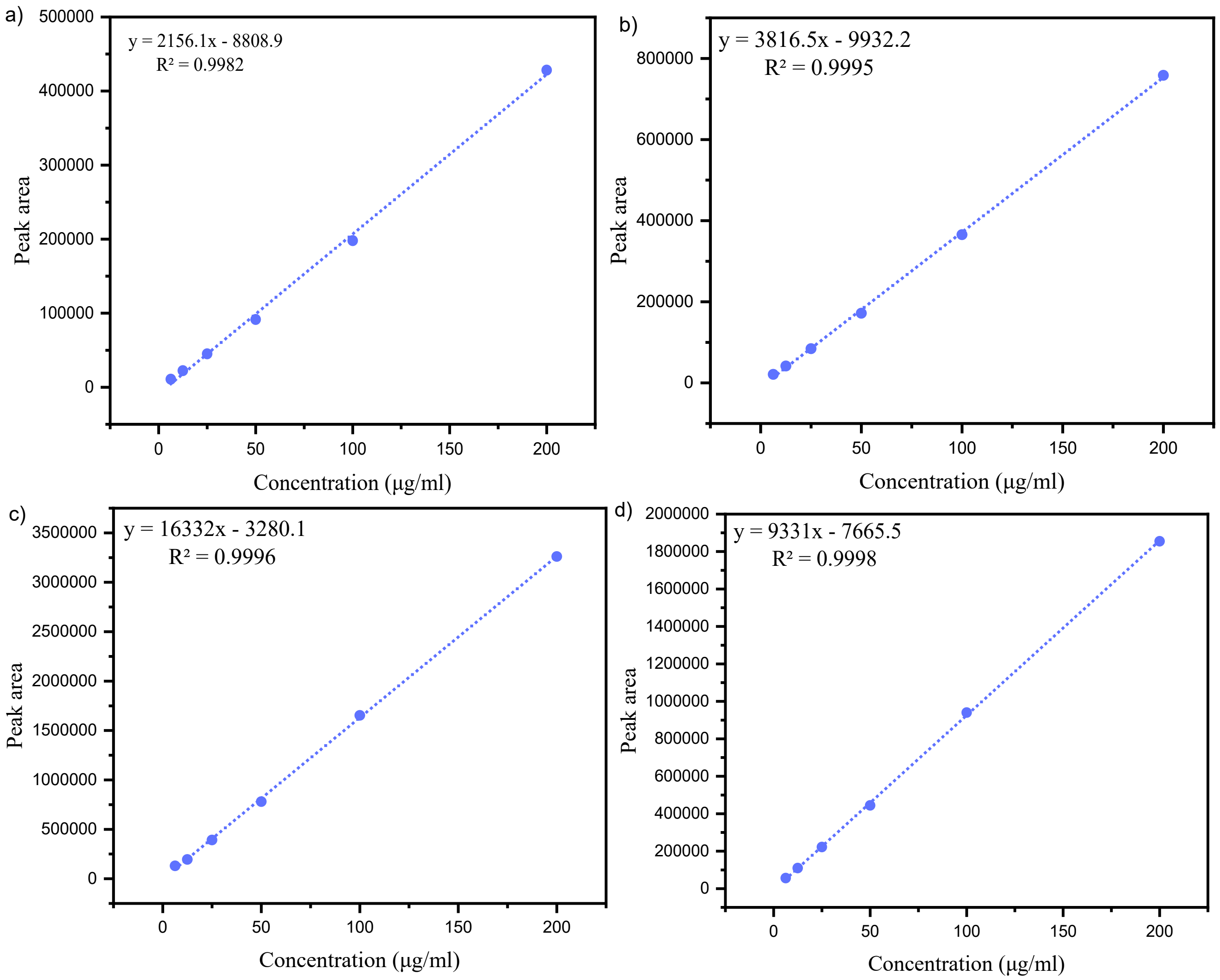

Figure S1. HPLC standard curve of the four reference alkaloids: a) protopine, b) allocryptopine, c) sagunarine, and d) chelerythrine.

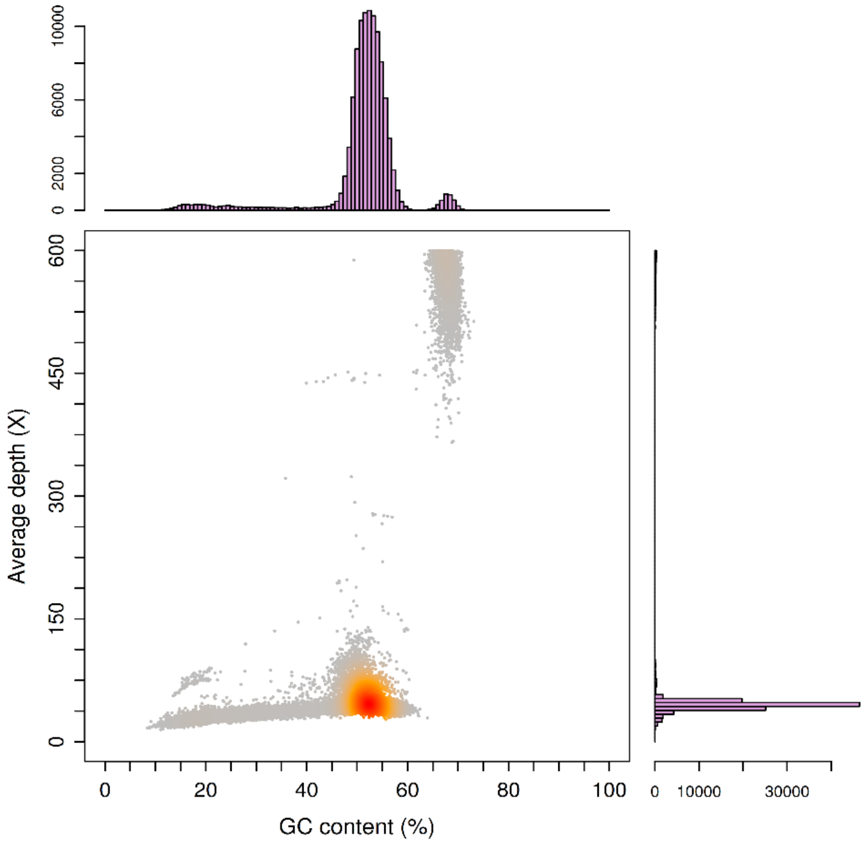

Figure S2. Guanine-cytosine content presence in the genomic data of the strain.

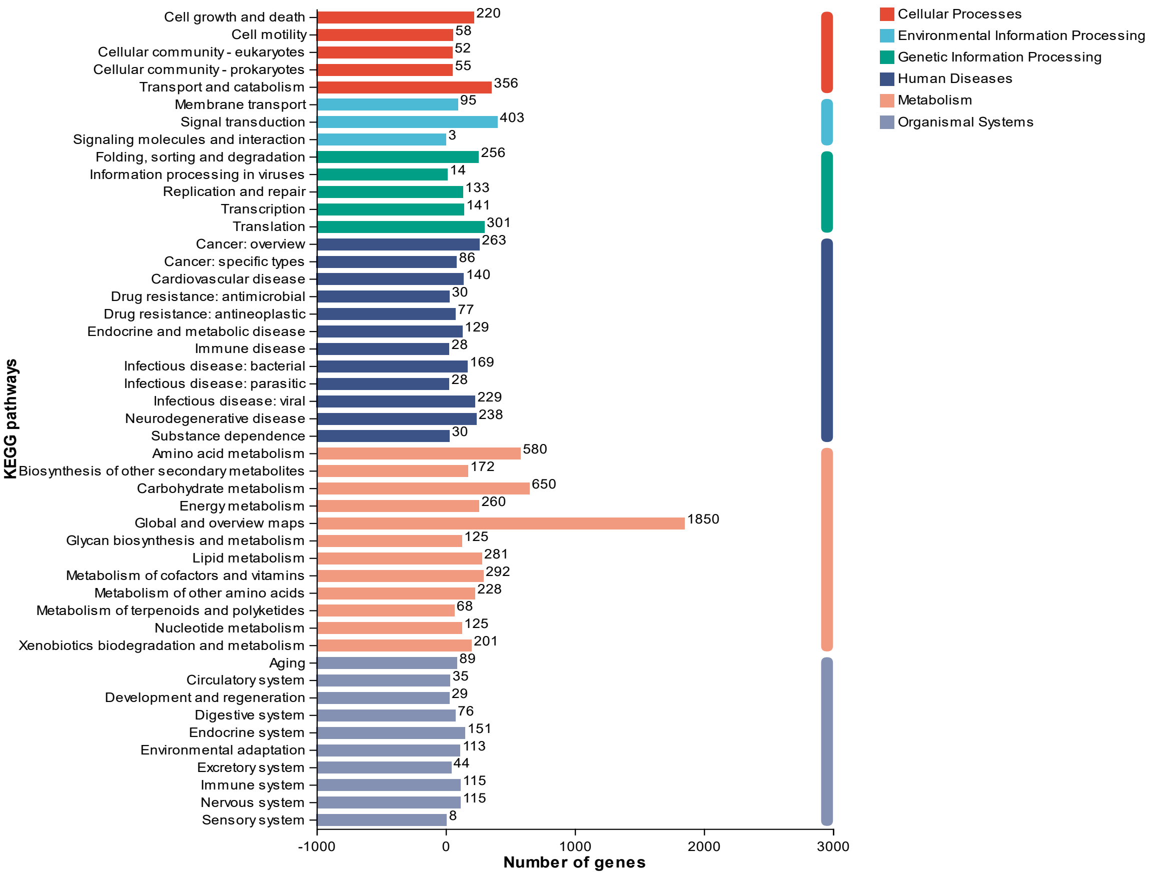

Figure S3. KEGG database annotation showing the enrichment of coding genes in six categories of pathways, where metabolism has the highest number of annotated genes (7955), comprised of amino acid metabolism (580), carbohydrates metabolism (650), etc.

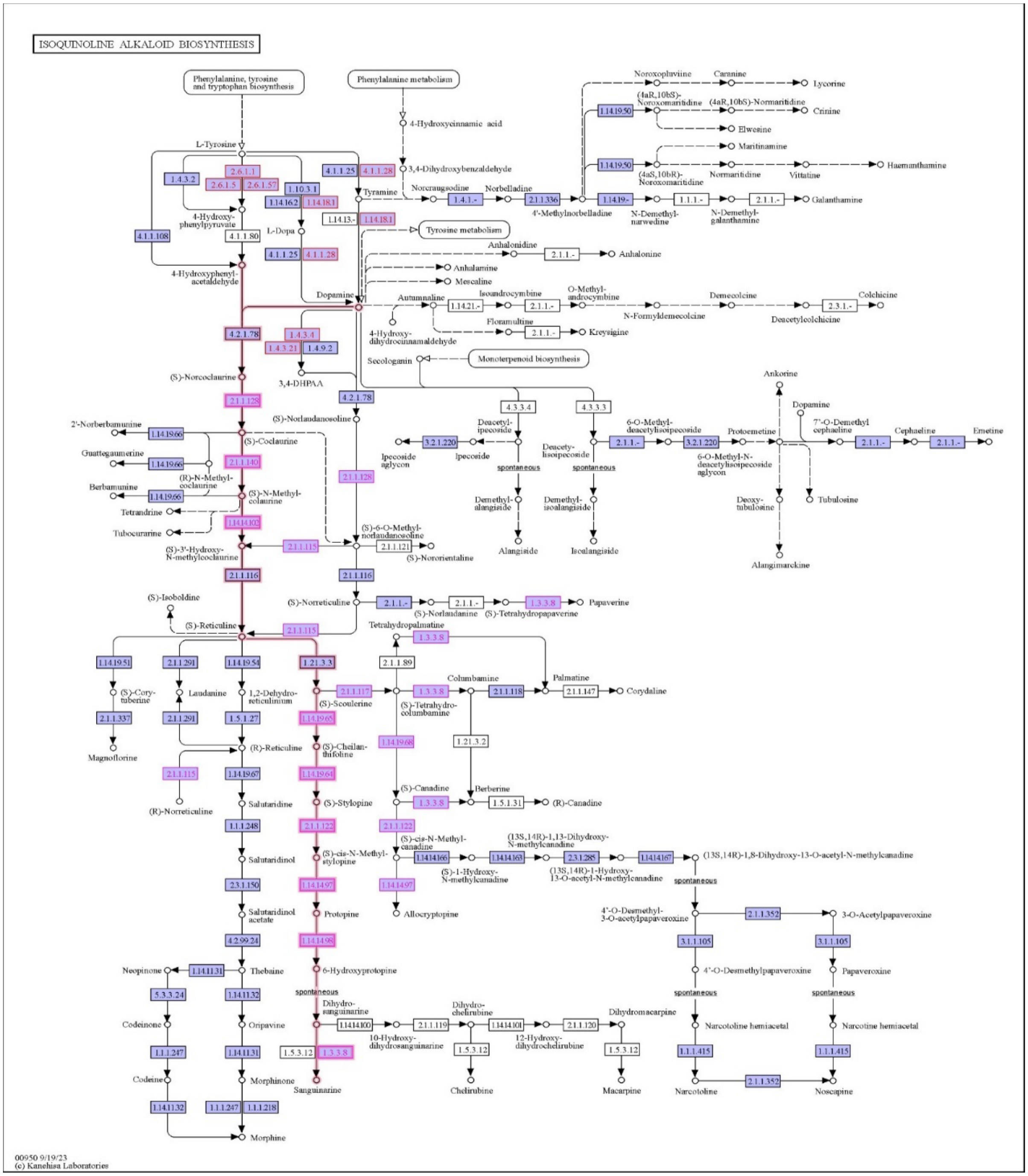

Figure S4. Isoquinoline alkaloids biosynthetic pathways from the KEGG database showing annotated genes from strain MC503 genome.

Table S1. Comparative analysis of sanguinarine and chelerythrine biosynthesis reference enzyme sequences against built local database sequences of the *Fusarium solani* strain MC503.

S/N	Enzyme Name	Gene ID	Align length	Identity (%)	Coverage (%)	Score	E-Value
1.	TYDC	gene07935	37	61.9	61.7	75	0.005121
		gene07935	38	53.5	63.3	72	0.012204
		gene07935	31	55.9	51.7	63	0.128499
		gene09279	24	56	40	67	0.045568
		gene07068	31	55.6	51.7	65	0.072227
		gene15871	34	50	56.7	65	0.091056
		gene04392	38	45.6	63.3	61	0.228876
		gene02110	37	37.8	61.7	57	0.616751
		gene11115	16	76.5	26.7	57	0.758257
		gene13564	29	53.3	48.3	56	0.953639
2.	NCS	-	-	-	-	-	-
3.	6OMT	gene11265	40	31.9	66.7	58	0.518348
4.	CNMT	gene12230	59	63.3	98.3	200	2.74E-20
		gene08530	58	39	96.7	107	5.28E-07
		gene01608	35	36.1	58.3	57	0.78739
5.	NMCH	gene10031	54	43.6	90	110	1.58E-07
		gene13865	30	50	50	82	0.000519
		gene14524	33	40	55	78	0.001815
		gene13992	16	64.7	26.7	71	0.013782
		gene09525	30	41.9	50	70	0.018649
		gene14543	28	51.7	46.7	61	0.256516
		gene16574	34	45.7	56.7	59	0.398457
6.	4OMT	-	-	-	-	-	-
7.	BBE	-	-	-	-	-	-
8.	CFS	gene10031	16	64.7	26.7	70	0.020739
		gene13865	49	28.8	81.7	57	0.836913
9	STS	gene10031	18	57.9	30	68	0.034777
		gene08950	43	40.9	71.7	60	0.2694
		gene14524	17	55.6	28.3	56	0.878885
10.	TNMT	gene05771	46	25.5	76.7	56	0.921744
11.	MSH	-	-	-	-	-	-
12.	P6H	gene17284	47	35.3	78.3	70	0.016503
13.	DBOX	gene01328	43	31.9	71.7	57	0.867258

Table S2. Annotated enzyme/genes and alkaloid profiles of *Fusarium solani* strain MC503

KO Number	Functional Enzyme/Gene	Metabolic reaction	
		Reactants	Products
K14455 [EC:2.6.1.1]	aspartate aminotransferase (GOT2)	L-Tyrosine/L-Phenylalanine	4-Hydroxyphenylpyruvate/ Phenylpyruvate
K00815 [EC:2.6.1.5]	tyrosine aminotransferase (TAT)	L-Tyrosine/L-Phenylalanine	4-Hydroxyphenylpyruvate/ Phenylpyruvate
K00832 [EC:2.6.1.57]	aromatic-amino-acid transaminase (tyrB)	L-Tyrosine	4-Hydroxyphenylpyruvate
K00505 [EC:1.14.18.1]	Tyrosinase (TRY)	L-Tyrosine	L-Dopa
K01593 [EC:4.1.1.28 4.1.1.105]	aromatic-L-amino-acid/L-tryptophan decarboxylase (DDC, TDC)	L-Tyrosine /L-Dopa/L-Tryptophan	Tyramine/Dopamine/Tryptamine
K00505 [EC:1.14.18.1]	Tyrosinase (TYR)	Tryptamine	Dopamine
K00274 [EC:1.4.3.4]	monoamine oxidase (MAO)	Dopamine	3,4-DHPAA
K00276 [EC:1.4.3.21]	primary-amine oxidase (AOC3, AOC2, tynA)	Dopamine/N-Methyl putrescine/Cadaverine	3,4-DHPAA/1-Methyl pyrrolinium/5-Aminopentanal
K13383 [EC:2.1.1.128]	(RS)-norcoclaurine 6-O-methyltransferase	(S)-Norcoclaurine	(S)-Coclaurine
K13384 [EC:2.1.1.115 2.1.1.140]	(RS)-1-benzyl-1,2,3,4-tetrahydroisoquinoline N-methyltransferase (CNMT)	(S)-Coclaurine	(S)-N-Methyl colaurine
K13385 [EC:1.14.14.102]	N-methylcoclaurine 3'-monooxygenase (CYP80B1)	(S)-N-Methyl colaurine	(S)-3′-Hydroxy-N-methylcoclaurine
K13383 [EC:2.1.1.128]	(RS)-norcoclaurine 6-O-methyltransferase	(S)-Norlaudanosoline	(S)-6-O-Methyl norlaodanosoline
K13384 [EC:2.1.1.115 2.1.1.140]	(RS)-1-benzyl-1,2,3,4-tetrahydroisoquinoline N-methyltransferase (CNMT)	(S)-6-O-Methyl norlaodanosoline	(S)-3′-Hydroxy-N-methylcoclaurine
K13384 [EC:2.1.1.115 2.1.1.140]	(RS)-1-benzyl-1,2,3,4-	(S)-Norreticuline	(S)-Reticuline

	tetrahydroisoquinoline N-methyltransferase (CNMT)		
K21070 [EC:1.14.19.65]	(S)-cheilanthifoline synthase (CYP719A14)	(S)-Scoulerine	(S)-Cheilanthifoline
K13395 [EC:1.14.19.64 1.14.19.68 1.14.19.73]	(S)-stylopine/(S)-canadine/(S)-nandinine synthase	(S)-Cheilanthifoline/ Tetrahydrocolumbamine	(S)-stylopine/(S)-Canadine
K13396 [EC:2.1.1.122]	(S)-tetrahydroprotoberberine N-methyltransferase (E2.1.1.122)	(S)-stylopine/canadine	(S)-cis-N-Methyl stylopine/(S)-cis-N-Methylcanadine
K21692 [EC:1.14.14.97]	methyltetrahydroprotoberberine 14-monooxygenase (CYP82N4)	(S)-cis-N-Methyl stylopine/(S)-cis-N-Methylcanadine	Protopine/Allocryptopine
K21693 [EC:1.14.14.98]	protopine 6-monooxygenase (CYP82N2_3)	Protopine	6-Hydroprotopine
K22089 [EC:1.3.3.8]	tetrahydroprotoberberine oxidase (STOX, DBOX)	(S)-Tetrahydropapaverine/Tetrahydropalmatine/(S)-Tetrahydrocolumbamine/ (S)-Canadine/ Dihydrosanguinarine	Papaverine/Palmatine / Columbamine/ Berberine / Sanguinarine
K13397 [EC:2.1.1.117]	(S)-scoulerine 9-O-methyltransferase (SMT)	(S)-Scoulerine	Tetrahydrocolumbamine
K00817 [EC:2.6.1.9]	histidinol-phosphate aminotransferase (hisC)	L-Phenylalanine	Phenylpyruvate
K00832 [EC:2.6.1.57]	aromatic-amino-acid transaminase (tyrB)	L-Phenylalanine	Phenylpyruvate
K08081 [EC:1.1.1.206]	tropinone reductase I (TR1)	Tropinone	Tropine
